# Outcomes of surgical management of carcinoid heart disease in patients with primary gonadal neuroendocrine tumors

**DOI:** 10.1016/j.xjon.2025.05.010

**Published:** 2025-06-10

**Authors:** Tedy Sawma, Hartzell V. Schaff, Anita Zheng, Gokce Belge Bilgin, Thorvardur R. Halfdanarson, S.Allen Luis, Patricia A. Pellikka, Heidi M. Connolly, Juan A. Crestanello

**Affiliations:** a Department of Cardiovascular Surgery, Mayo Clinic, Rochester, Minn; b Department of Radiology, Mayo Clinic, Rochester, Minn; c Department of Oncology, Mayo Clinic, Rochester, Minn; d Department of Cardiovascular Medicine, Mayo Clinic, Rochester, Minn

**Keywords:** ovarian carcinoid tumor, ovarian neuroendocrine tumor, testicular neuroendocrine tumor, carcinoid heart disease, tricuspid valve replacement

## Abstract

**Objectives:**

To describe the clinical presentation of patients with gonadal neuroendocrine tumors and carcinoid heart disease (CaHD) and to evaluate long-term outcomes following valvular surgery.

**Methods:**

Retrospective review of patients with primary gonadal neuroendocrine tumor who were surgically treated for CaHD at our institution between 1990 and 2021.

**Results:**

Eight patients (median age, 70 years) were included in the study, 7 with ovarian tumors and 1 with testicular tumor. None of the patients had liver metastasis at the time of cardiac surgery. Three patients presented with both CaHD symptoms and carcinoid syndrome symptoms (diarrhea and flushing). Three others presented with symptoms of CaHD but without diarrhea or flushing. One patient with ovarian tumor presented with severe diarrhea and flushing without CaHD symptoms and had tumor resection but then developed severe CaHD symptoms few months later. The last patient presented initially with an asymptomatic testicular mass, which was resected, but then developed severe CaHD symptoms years later. All patients had severe tricuspid regurgitation at time of surgery, and 7 had severe pulmonary regurgitation. All were treated with replacement of affected valves. Both 5- and 10-year survival rates were 86% and were higher than a control group of patients with CaHD and nongonadal primary neuroendocrine tumor (35% and 23%, respectively).

**Conclusions:**

Patients with primary gonadal neuroendocrine tumors can develop CaHD in the absence of liver metastasis. Some patients have delayed presentation of cardiac symptoms, emphasizing the importance of thorough assessment and regular echocardiographic follow-up. Cardiac intervention is safe and yields excellent long-term survival.

## Graphical abstract

**Figure:**
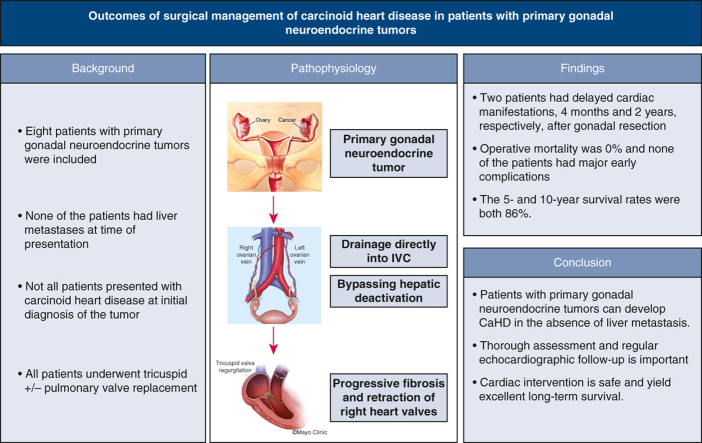

**Figure:**
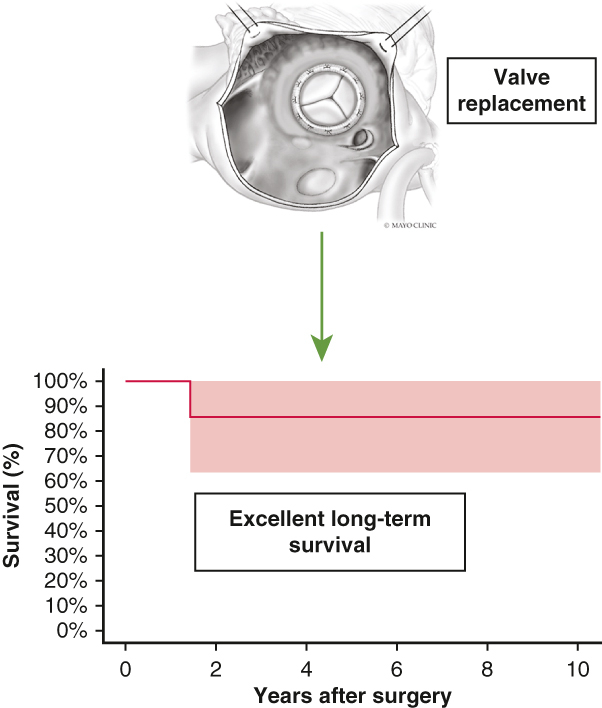
Valve replacement in patients with CaHD due to primary gonadal tumors is associated with excellent long-term survival.

**Central Message**

Timely surgical intervention for carcinoid heart disease in patients with primary gonadal neuroendocrine tumors is associated with excellent late survival outcomes.

**Perspective**

Primary gonadal neuroendocrine tumors can lead to carcinoid heart disease in the absence of liver metastasis. Some patients do not present with cardiac symptoms initially, only to develop delayed-onset cardiac manifestations months or years after resection of the primary tumor. This highlights the importance of comprehensive cardiac screening at time of initial diagnosis and close clinical follow-up.

Primary gonadal neuroendocrine tumors represent a rare subset of neuroendocrine neoplasms accounting for <0.5% of all neuroendocrine tumors.^1^, ^2^, ^3^ Unlike primary tumors of the small bowel and other intra-abdominal sites, tumors of gonadal origin can cause carcinoid heart disease (CaHD) and subsequent right ventricular (RV) heart failure in the absence of liver metastasis.^4^^,^^5^ This unique mechanism is attributed to the venous drainage of the gonads directly into the inferior vena cava, allowing tumor-secreted serotonin to enter the systemic venous circulation while bypassing metabolic deactivation by the liver.^6^^,^^7^

In this study, we review our experience in managing patients with primary gonadal neuroendocrine tumors complicated by CaHD. The study aims to highlight the variability in clinical presentations at initial diagnosis and to evaluate long-term outcomes following valvular surgery.

## Material and Methods

### Study Design

The Mayo Clinic Institutional Review Board, Rochester, MN, approved the study (No. 23-010347) on December 22, 2023. Although the institutional review board waived the requirement for specific patient consent for this study, all patients undergoing surgery at our institution are asked to provide written authorization for the use of their clinical data in future research. Only patients who have granted this authorization are included in research studies. Between 1990 and 2021, 8 patients with CaHD and a primary ovarian or testicular neuroendocrine tumor were examined and managed surgically at Mayo Clinic.

### Data Collection and Study Group

Relevant demographic characteristics, comorbidities, imaging data, operative data, immediate postoperative outcomes, and long-term outcomes were collected manually. Comprehensive resting 2-dimensional transthoracic echocardiography was performed in all patients before cardiac surgery and was in accordance with the guidelines of the American Society of Echocardiography.^8^

**Figure 1:**
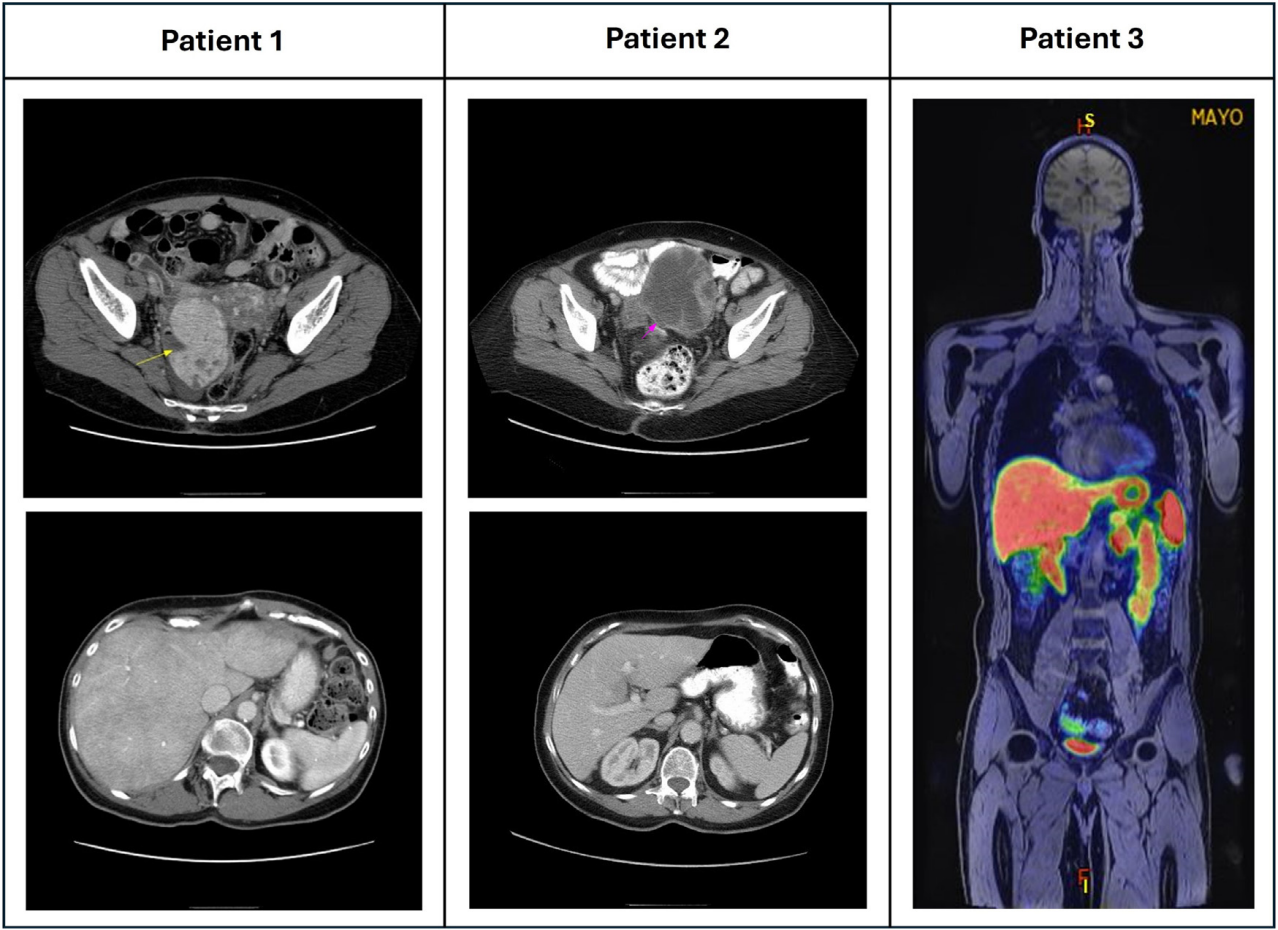
Computed tomography scans (*axial view*) of the ovarian tumors and nonaffected liver for the same individual at the time of cardiac surgery (patients 1 and 2). Gallium-68–labeled peptide positron-emission tomography scan showing a healthy liver in the patient with testicular tumor (resected at time of cardiac surgery).

The diagnosis of primary gonadal neuroendocrine tumor was confirmed by a combination of imaging and immunohistochemistry. For cases referred from external institutions, biopsies underwent a secondary review to validate the diagnosis pathologically. Gallium-68–labeled peptide positron-emission tomography was prioritized when available, or else computed tomography scan and magnetic resonance imaging images were used to visualize the ovarian mass and to look for distant metastases (Figure 1). The primary end point of the study was long-term all-cause mortality. A control group consisting of patients with CaHD and a primary nongonadal neuroendocrine tumor was used qualitatively as a comparative group to illustrate differences in long-term survival.

### Cardiac Operative Method

The surgical technique for CaHD in patients with primary gonadal tumors does not differ from that for patients with nongonadal primary tumors. In brief, the majority of patients with CaHD exhibit some degree of tricuspid valve regurgitation, and most will require tricuspid valve replacement.^9^ In our early practice, the pulmonary valve was often excised during tricuspid valve replacement. Although early postoperative outcomes following pulmonary valvectomy were generally favorable, long-term follow-up revealed an increased risk of progressive RV dilation.^10^ As a result, our current approach is to replace the pulmonary valve in patients presenting with severe pulmonary valve regurgitation. Prophylactic replacement of the pulmonary valve for patients with less-than-severe regurgitation is not needed since the risk of progression into severe regurgitation is low.^10^

Careful preoperative planning and perioperative management were implemented for patients with CaHD to reduce the risk of life-threatening carcinoid crises and enable prompt intervention if a crisis occurred intraoperatively. Hemodynamic instability related to serotonergic secretions was managed intraoperatively through the use of intravenous octreotide acetate and vasopressors. Additionally, aggressive management, including hemofiltration and diuresis, were instituted for patients with long-standing right-sided heart failure.

For patients undergoing concomitant gonadal resection, the procedure was performed after completion of the cardiac surgery. The patient was repositioned, then prepped and draped in the usual sterile fashion. A lower midline incision was made to enter the peritoneal cavity and carry out the gonadal resection.

### Statistical Analysis

Categorical data are presented as frequencies and percentages and continuous variables as medians and interquartile ranges (IQRs). Long-term survival was estimated using the Kaplan-Meier method. We included a survival curve for the general surgical CaHD population at our clinic to allow for an informal comparison of outcomes. Ninety-five percent confidence bands for both curves were also included. Given the limited sample size of 8 patients, survival curves were not truncated.

## Results

### Patient Characteristics

**Figure 2:**
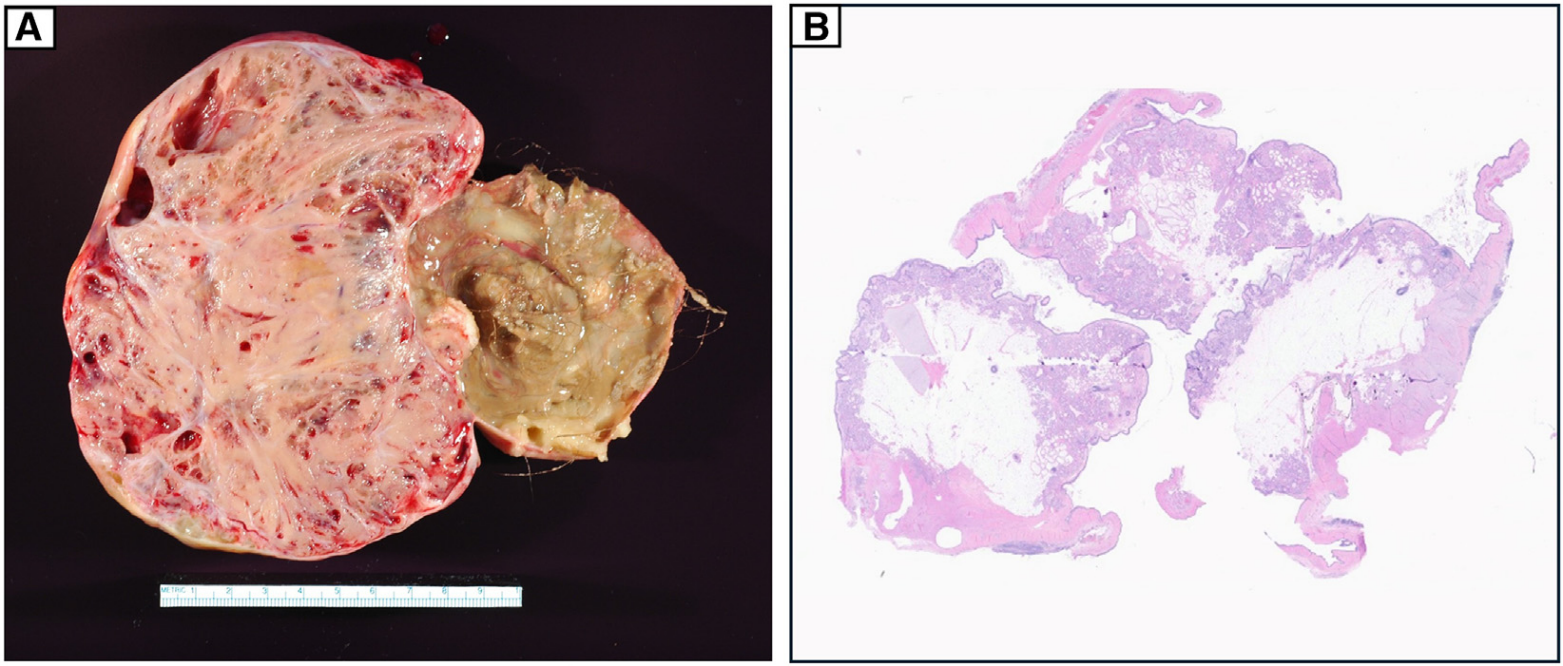
Ovarian neuroendocrine tumor arising from a mature cystic teratoma. A, Surgical view. B, Cytology.

Eight patients (median age, 70 years [IQR, 50-75 years]) with primary gonadal neuroendocrine tumors underwent valvular surgery for CaHD (Table 1). Seven had ovarian tumors and 1 had a testicular tumor. The surgical pathology of an ovarian tumor was provided in Figure 2. None of the patients had metastasis to the liver (Figure 1), 1 had metastasis to the lung and distant lymph nodes, and 2 others had metastases to distant lymph nodes only.

**Table 1:** Baseline characteristics and echocardiographic parameters (N = 8)

Variable	Result	n
Baseline characteristic		
Age (y)	71 (44-76)	8
BMI	25 (22-31)	7
Creatinine (mg/dL)	1.0 (0.9-1.1)	7
Female	7 (87.5)	8
NYHA functional class III or IV	5 (62.5)	8
Use of diuretics	5 (71.4)	7
Preoperative somatostatin	4 (50)	8
Diabetes mellitus	2 (25)	8
Site of metastasis		8
Distant LNs	3 (25)	
Lung	1 (12.5)	
Bone	0 (0)	
Liver	0 (0)	
Urine 5-HIAA (mg/24 h)	88 (25-131)	7
Echocardiographic parameters		
Severe tricuspid valve regurgitation	8 (100)	8
Tricuspid valve stenosis	2 (25)	8
Severe pulmonic valve regurgitation	7 (87.5)	8
Pulmonic valve stenosis	3 (37.5)	8
Right ventricular systolic pressure (mm Hg)	40 (35-54)	6
Right ventricular dysfunction		8
Normal	2 (25)	
Mild	1 (12.5)	
Moderate	4 (50)	
Severe	1 (12.5)	
Right ventricular enlargement		8
Normal	1 (12.5)	
Mild	1 (12.5)	
Moderate	0 (0)	
Severe	6 (75)	
Left ventricular ejection fraction (%)	59 (46-60)	8

Values are presented as n (%) or median (interquartile range).

*BMI*, Body mass index; *NYHA*, New York Heart Association; *LN*, lymph node; *5-HIAA*, 5-hydroxyindoleacetic acid.

### Presentation of Carcinoid Syndrome

Three patients presented with severe symptoms of carcinoid syndrome, including diarrhea and flushing, in addition to symptoms of CaHD (ie, dyspnea, fatigue, and leg edema) and were found to have ovarian masses on imaging. Two of these patients underwent concomitant valvular surgery and laparotomy for tumor resection. The third had valve replacement due to severe heart failure symptoms and subsequently underwent laparotomy 4 months later.

Three other patients presented with symptoms of heart failure related to CaHD without experiencing diarrhea or flushing. Further evaluation revealed ovarian masses consistent with primary gonadal neuroendocrine tumors. In 2 cases where the cardiac symptoms were mild, the patients initially underwent tumor resection, which led to improvements in their nutritional status and level of activity. However, both required cardiac surgery a few months later due to progressive worsening of heart failure symptoms. In the third case, the patient underwent cardiac surgery first due to severe symptoms and was subsequently followed up at a different clinic.

One patient initially presented with severe diarrhea and flushing without CaHD symptoms and was found to have a neuroendocrine ovarian tumor, which was resected at a different center. Echocardiographic assessment was not performed initially. The patient was then referred to our Clinic 4 months later with severe RV heart failure symptoms and was diagnosed with CaHD, necessitating cardiac surgery.

The last patient initially presented with an asymptomatic testicular mass that was resected and diagnosed as a neuroendocrine tumor upon pathological assessment. Two years later, the patient was referred to our clinic with severe heart failure symptoms requiring valvular intervention. It should be noted that a chest computed tomography scan with contrast showed normal heart and great vessel appearance at time of initial presentation of the testicular mass 2 years ago, but no echocardiographic assessment was performed at that time. Additionally, the patient had no evidence of tumor recurrence at the time of cardiac surgery.

### Presentation at the Time of Cardiac Surgery

**Figure 3:**
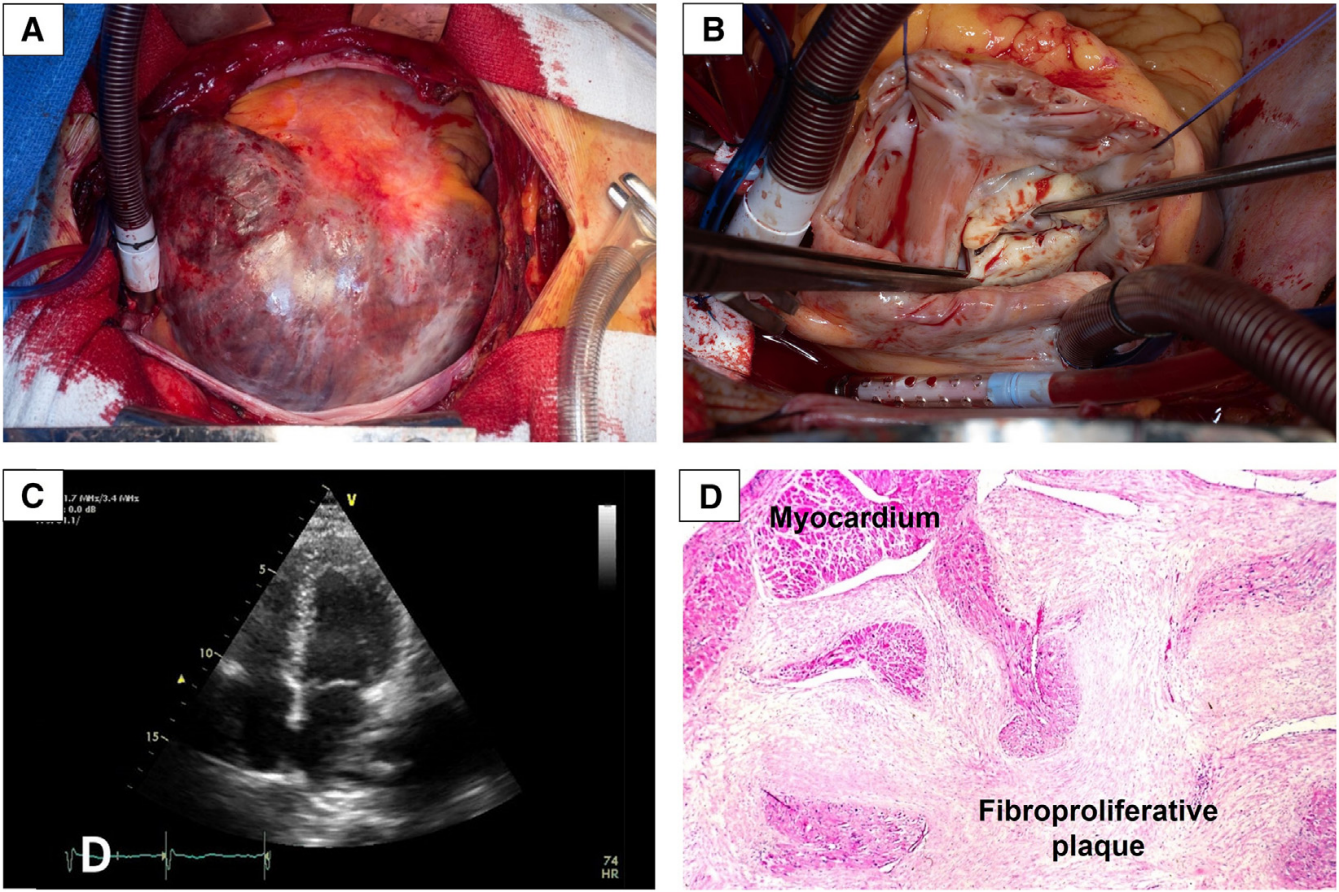
A, Prominent right atrial enlargement on surgical view. B, Thickened and retracted tricuspid valve cusps. C, Four-chamber view on echocardiography showing right atrial and ventricular enlargement as well as thickening and retraction of tricuspid valve cusps. D, Histopathology of resected tricuspid valve cusp showing thickening due to fibroproliferative plaque formation.

Advanced heart failure symptoms (New York Heart Association functional class III or IV) were present in 5 patients (62.5%). All patients had severe tricuspid valve regurgitation, and 7 (87.5%) had severe pulmonary valve regurgitation. Only 1 patient had moderate mitral valve regurgitation. None had aortic valve regurgitation. Right heart disease and valvular pathology are further illustrated in Figure 3.

Tricuspid valve replacement was performed in all the patients and pulmonic valve replacement in 6. For the tricuspid valve, 7 patients received a bioprosthetic valve and 1 received a mechanical valve. Among patients having pulmonary valve replacement, 5 had a bioprosthetic valve and 1 had a mechanical valve. One patient had pulmonary valvectomy. None of the patients needed left heart valvular surgery.

### Early and Late Postoperative Outcomes

None of the patients died during their hospital stay. Median intensive care unit stay was 28 hours (IQR, 24-44 hours) and median hospital stay was 6 days (IQR, 5-8.5 days). One patient needed pacemaker implantation due to complete heart block following surgery. Further details are provided in Table 2.

**Table 2:** Operative details and postoperative outcomes

Operative details	Result	n
Tricuspid valve replacement		8
Bioprosthetic	7 (87.5)	
Mechanical	1 (12.5)	
Pulmonic valve surgery		8
Replacement	6 (75)	
Valvectomy	1 (12.5)	
None	1 (12.5)	
Mitral valve replacement	0 (0)	8
Aortic valve replacement	0 (0)	8
Bypass time (min)	80 (66-96)	8
Concomitant patent foramen ovale repair	2 (25)	8
Timing of gonadal tumor resection		8
Before cardiac surgery	4 (50)	
After cardiac surgery	2 (25)	
With cardiac surgery	2 (25)	
30-d Mortality	0 (0)	8
Length of hospital stay (d)	6 (5-8)	8
Length of ICU stay (h)	26 (24-44)	8
Postoperative atrial fibrillation	0 (0)	8
Postoperative stroke	0 (0)	8
Reoperation for bleeding	0 (0)	8
Need for cardiac implantable device (pacemaker/ICD)	1 (12.5)	8
Congestive heart failure	0 (0)	8
Need for dialysis	0 (0)	8

Values are presented as n (%) or median (interquartile range).

*ICU*, Intensive care unit; *ICD*, implantable cardioverter defibrillator.

**Figure 4:**
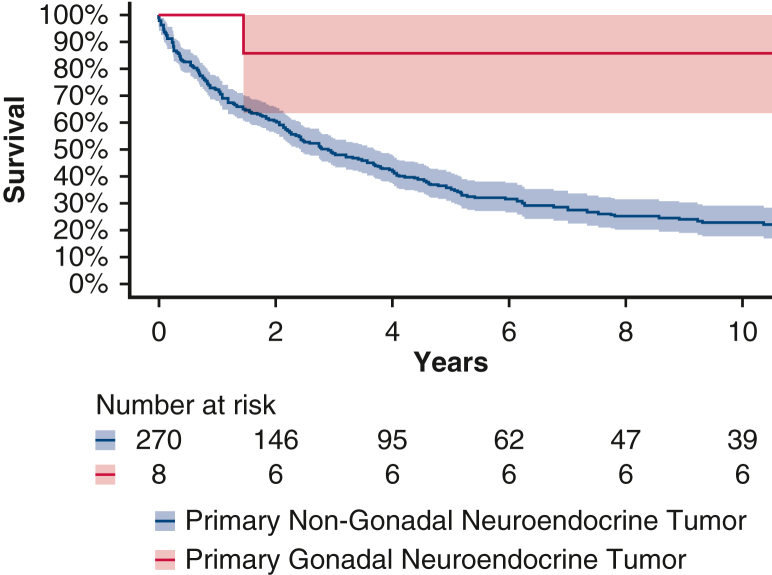
Kaplan-Meier survival curves for patients with primary gonadal neuroendocrine tumor undergoing valvular surgery versus the overall surgical carcinoid heart disease population at our clinic. 95% CI. *CHD*, Carcinoid heart disease.^17^

The 5- and 10-year survival rates were 86% (95% CI, 63%-100%) in patients with CaHD with primary gonadal tumors. In contrast, the 5- and 10-year survival rates were 35% (95% CI, 30%-42%) and 23% (95% CI, 18%-29%), respectively, in patients with primary nongonadal tumors. These survival rates are further illustrated in Figure 4.

## Discussion

Gonadal neuroendocrine tumors are a rare type of gonadal neoplasms that can cause CaHD even in the absence of hepatic metastasis, due to the anatomical drainage of the gonads directly into the inferior vena cava.^11^ This study represents the largest single-center series to comprehensively address the presentation and management of CaHD in patients with primary neuroendocrine tumors of the gonads.

Serotonin is widely recognized as a key factor in the progression of CaHD.^12^ However, in our series, we observed that some patients with ovarian neuroendocrine tumors did not present with symptoms of CaHD at initial diagnosis but later developed severe symptoms and RV failure despite resection of the primary tumor. Progression into CaHD suggests that these patients may have mild valvular involvement that was either undetectable/subclinical or missed during the initial workup, particularly in cases where baseline echocardiographic assessment was not performed. The initial serotonergic secretion from the primary tumor appears to cause irreversible damage or scarring to the cardiac valves, with inevitable progression into severe valvular regurgitation and RV heart failure, and this has been reported in a single case in the literature.^13^ Such findings underscore the importance of comprehensive cardiac screening at time of initial diagnosis and close clinical follow-up for all patients diagnosed with primary ovarian neuroendocrine tumors,^14^, ^15^, ^16^ even in the absence of any cardiac symptoms or echocardiographic evidence at baseline.

In patients presenting initially with CaHD, a concomitant abdominal surgery at the time of valvular replacement seems to be a safe option. Indeed, 2 patients in our study had simultaneous cardiac surgery and resection of their primary ovarian tumor without any complications during their postoperative hospital stay. This is in accordance with previous studies from the literature reporting acceptable outcomes in patients with concomitant cardiac and noncardiac surgeries, including thoracic, abdominal, and vascular procedures.^17^, ^18^, ^19^, ^20^, ^21^

**Figure 5:**
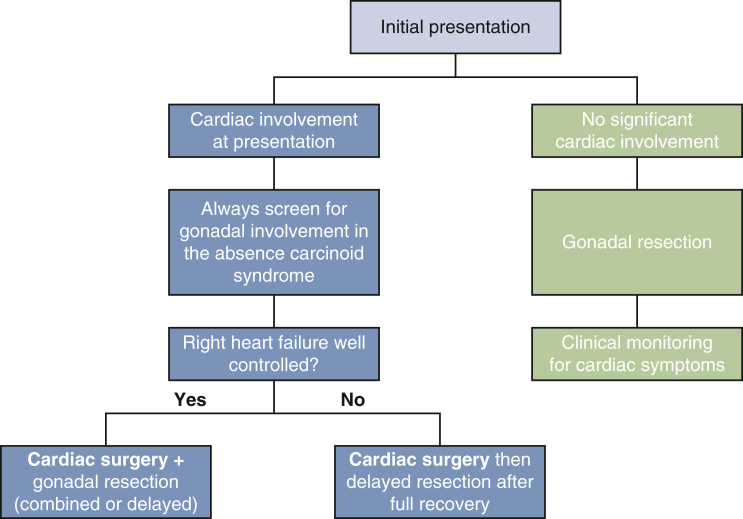
Suggested treatment plan for patients with gonadal neuroendocrine tumors presenting with or without carcinoid heart disease.

The decision regarding the sequence of cardiac and gynecologic procedures depends on the patient's condition and the surgical teams' experience. If the patient's right heart failure is well controlled, valve replacement and excision of the gonadal tumor can be performed during the same general anesthetic, thus sparing the patient 2 separate hospitalizations. If right heart failure is a prominent clinical feature, cardiac surgery should be performed first, and the gynecologic procedure should be deferred until full recovery. The 2 patients in our series who underwent gynecologic resection before cardiac surgery had only mild cardiac symptoms, and both their diagnosis and initial surgical management were performed at outside institutions before being referred to our center for cardiac intervention. Our recommendations are further illustrated in Figure 5.

The long-term survival of patients with primary ovarian neuroendocrine tumors is significantly better than that of patients with CaHD with intra-abdominal or intrathoracic primary neuroendocrine tumors. In our series, the 5-year survival rate was 86%, compared with 30% to 35% in the general surgical CaHD population reported in the literature.^22^ A study conducted at our institution assessing the influence of tumor burden on survival in patients with CaHD undergoing cardiac surgery found that the extent of metastatic liver involvement and the presence of bone metastasis were major factors associated with poor postoperative survival (unpublished data). This may explain the better survival rates observed in patients with CaHD with primary ovarian tumors, who have significantly lower tumor burden at the time of valvular intervention. Notably, the 5-year survival rate of patients with CaHD with primary ovarian tumors following cardiac intervention (86%) is comparable to that of patients with isolated (stage I) ovarian neuroendocrine tumors without cardiac involvement (83.3%), as reported by Pang and colleagues^22^ using the Surveillance, Epidemiology, and End Results database. Therefore, timely surgical intervention on the tricuspid and pulmonary valves in these patients appears to significantly improve long-term survival, in contrast to the outcomes seen in patients with intra-abdominal primary tumors where postoperative survival is limited due to extensive tumor progression.

### Limitation

This is a single-center study with small sample size limiting generalizability of the results. Nonetheless, being a referral center of CaHD, we were able to present different cases of a rare disease presentation, which may provide valuable diagnostic and therapeutic guidance for centers with less familiarity with the management of CaHD.

## Conclusions

Patients with primary neuroendocrine tumor of the gonads can develop CaHD in the absence of metastatic liver involvement. Some patients may have delayed presentation of cardiac symptoms prompting comprehensive diagnostic assessment at initial presentation in addition to close clinical follow-up. Cardiac intervention is safe and is associated with excellent long-term survival outcomes.

## Conflict of Interest Statement

The authors reported no conflicts of interest.

The *Journal* policy requires editors and reviewers to disclose conflicts of interest and to decline handling or reviewing manuscripts for which they may have a conflict of interest. The editors and reviewers of this article have no conflicts of interest.
